# Ibuprofen Does Not Prevent Inhibition of Fetal Breathing Movements Caused by Intrauterine Inflammation in Fetal Sheep

**DOI:** 10.3390/ijms26125591

**Published:** 2025-06-11

**Authors:** Nhi T. Tran, Vanesa Stojanovska, Sharmony B. Kelly, Kayla Vidinopoulos, John Atta, Eva Matthews-Staindl, Valerie A. Zahra, Yen Pham, Eric A. P. Herlenius, Stuart B. Hooper, Beth J. Allison, Robert Galinsky, Graeme R. Polglase

**Affiliations:** 1 The Ritchie Centre, Hudson Institute of Medical Research, Clayton, VIC 3168, Australia; nhi.tran@hudson.org.au (N.T.T.);; 2 Department of Obstetrics and Gynaecology, Monash University, Clayton, VIC 3168, Australia; 3 Department of Women’s and Children’s Health, Karolinska Institutet, 17176 Solna, Sweden; 4 Astrid Lindgren Children’s Hospital, Karolinska University Hospital Stockholm, 17176 Solna, Sweden; 5 Department of Pediatrics, Monash University, Clayton, VIC 3168, Australia

**Keywords:** fetal breathing movements, intrauterine inflammation, PGE_2_, brainstem, apnoea, preterm brain

## Abstract

Antenatal inflammation/infection is a major cause of neonatal apnoea and hypoventilation. Prostaglandin E2 (PGE_2_) is a key inflammatory mediator associated with depression of fetal and neonatal breathing. We aimed to determine whether antenatal ibuprofen, a cyclooxygenase inhibitor that reduces synthesis of PGE_2_, restores fetal breathing movements (FBM) in late-gestation fetal sheep exposed to systemic lipopolysaccharide (LPS). Fetal sheep (125 days gestation, d; term ~148 d) were instrumentally monitored for continuous measurement of FBM and physiological parameters. At 130 d fetuses were randomly allocated between groups receiving i.v. saline (CTL_SAL_, n = 9), escalating doses of LPS (i.v.) over 3 days (LPS_SAL_, n = 8), or ibuprofen one hour after each LPS dose (LPS_IBU_, n = 8). Regular plasma samples were collected for PGE_2_ assessment. At 135 d, cerebrospinal fluid and brainstem tissue were collected at autopsy for assessments of PGE_2_ expression, and immunohistochemical quantification of astrocytes and microglia within key brainstem respiratory centres was performed to assess inflammation. LPS exposure increased PGE_2_ levels in plasma, cerebrospinal fluid and the RTN/pFRG (*p* < 0.05) and decreased the incidence, amplitude and amount of the accentuated (>5 mmHg) FBMs. Ibuprofen reduced plasma and RTN/pFRG PGE_2_ expression (*p* < 0.01 and *p* = 0.031, respectively) but did not restore FBMs. Astrocyte and microglial density increased in the RTN/pFRG, NTS and raphe nucleus in LPS_IBU_ fetuses, compared to LPS_SAL_ (*p* < 0.05). Antenatal ibuprofen treatment did not restore depressed FBM, despite reducing the circulating and brainstem PGE_2_ levels in LPS-exposed fetal sheep. Other inflammatory pathways or more specific targeting of PGE_2_ may be more effective in preventing apnoea caused by exposure to intrauterine infection/inflammation.

## 1. Introduction

Preterm infants exposed to antenatal infection/inflammation often suffer from respiratory depression and subsequently have increased risks of apnoea and hypoxemia, and in some cases, require prolonged respiratory support [1,2,3,4]. One way to reduce respiratory depression and subsequently improve neonatal outcomes would be to stimulate spontaneous breathing.

Ibuprofen is an FDA-approved drug used clinically for the treatment of patent ductus arteriosus in newborn infants [5]. The mechanism of action of this non-steroidal anti-inflammatory drug involves the non-selective inhibition of cyclooxygenases 1 and 2 (COX1 and COX2), thereby decreasing the synthesis of prostaglandins such as prostaglandin E2 (PGE_2_). PGE_2_ is notably involved in the regulation of breathing during the perinatal period [6,7].

The brainstem is made up of a complex network of respiratory centres comprised of densely interconnected neurons that govern breathing, and is highly sensitive to PGE_2_ [8]. For example, systemic infusion of PGE_2_ causes marked inhibition of breathing movements (FBMs) in fetal sheep [9,10,11], and apnoea and altered breathing rhythmicity in mice [12]. Moreover, newborn infants with elevated PGE_2_ in their cerebrospinal fluid have increased apnoea frequency [3]. Intravenous infusion of lipopolysaccharide (LPS) to fetal sheep increases PGE_2_ within the circulation, cerebrospinal fluid and brainstem, and results in depressed FBMs [13]. These findings suggests that regulation of central PGE_2_ levels is a potential target for the restoration of breathing after infection/inflammation-induced respiratory depression.

The aim of this study was to assess whether ibuprofen could prevent the inhibition of FBMs in late-gestation fetal sheep exposed to LPS-induced inflammation. We hypothesised that intravenous injection of ibuprofen after LPS exposure would reduce PGE_2_ concentration in the circulation and brainstem respiratory centres and restore FBMs.

## 2. Results

### 2.1. Fetal Characteristics: Fetal Breathing Movement Characterisation

Fetal characteristics at post-mortem (135± d) are presented in Table 1. LPS_IBU_ fetuses had increased heart weight compared to CTL_SAL_ and LPS_SAL_ fetuses (*p* = 0.0019 and *p* = 0.0061, respectively) and a higher heart/body ratio compared to CTL_SAL_ fetuses (*p* = 0.015). There were no differences in body, brain, lung, liver, and right kidney weight between groups.

### 2.2. Fetal Breathing Movement Characterisation

At baseline, one hour (h) prior to the first LPS/saline infusion, all groups had similar FBM characteristics (incidence, duration, frequency, amplitude, and % of time spent exhibiting accentuated breathing) (Table 2).

**Figure 1:**
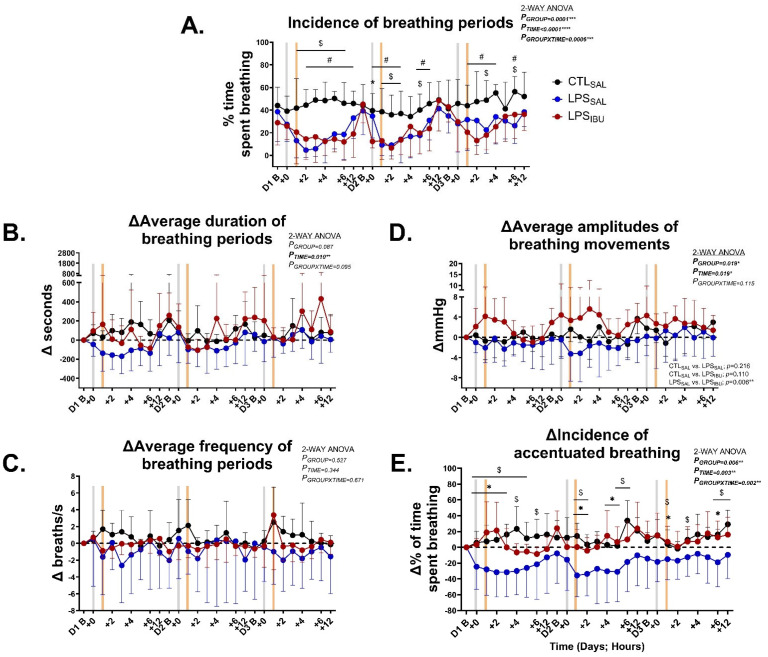
Fetal breathing movement (FBM) characterisations of (**A**) incidence (% of time spent breathing per h), (**B**) delta change from day 1 baseline (Δ) for the average duration of continuous breathing per h, (**C**) Δ change from baseline for average frequency of continuous breathing per h, (**D**) Δ change from baseline for average amplitudes of breaths per h, and (**E**) Δ change from baseline for incidence of accentuated breathing (% of time spent breathing per h). Hourly epochs were analysed throughout experimental days (**D**) 1–3 at baseline (before LPS/saline infusions [grey bar]) and throughout the first 12 h after LPS/saline infusions. The orange bar denotes the timing of saline or ibuprofen treatment, which was at 1 h after the LPS/saline infusion. Values are mean ± SD. CTL_SAL_: *n* = 9; LPS_SAL_: *n* = 8; LPS_IBU_: *n* = 8. Significant group differences were assessed by Fisher’s LSD test for multiple comparisons and are indicated as follows: CTL_SAL_ vs. LPS_SAL_: ^$^ *p* < 0.05; CTL_SAL_ vs. LPS_IBU_: *^#^ p* < 0.05; LPS_SAL_ vs. LPS_IBU_: * *p* < 0.05.

The incidence of FBMs significantly decreased in LPS_SAL_ fetuses between 1 and 6 h after the first LPS infusion, 1–3 and 5 h after the second LPS infusion, and 3 and 6 h after the third LPS infusion (all *p* < 0.05; Figure 1A). FBM were inhibited in LPS_IBU_ fetuses between 2 and 12 h after the first LPS infusion, 0–3 and 5–6 h after the second LPS infusion, and 1–4 and 6 h after the third LPS infusion (all *p* < 0.05; Figure 1A). At the onset of the second LPS infusion, FBM were significantly lower in LPS_IBU_ fetuses compared to both LPS_SAL_ and CTL_SAL_ fetuses (*p* = 0.013 and 0.0005, respectively; Figure 1A).

The average duration of breathing periods and average frequency of breathing periods were similar between groups (Figure 1B,C). The amplitude of FBMs in LPS_IBU_ fetuses was higher than in LPS_SAL_ fetuses (Figure 1D; *p* = 0.006). The amplitude of FBMs was similar between CTL_SAL_ and LPS_SAL_ fetuses. During FBMs, the proportion of accentuated breathing was significantly lower in LPS_SAL_ fetuses intermittently between 0 and 6 h after each LPS infusion, compared to CTL_SAL_ and LPS_IBU_ fetuses (all *p* < 0.05; Figure 1E). There was no difference between the CTL_SA_L and LPS_IBU_ groups.

### 2.3. Systemic Hemodynamics

All groups had similar baseline carotid blood flow (CBF), cerebral oxygen delivery (CDO_2_), mean arterial blood pressure (MABP) and fetal heart rate (FHR) (Table 2).

**Figure 2:**
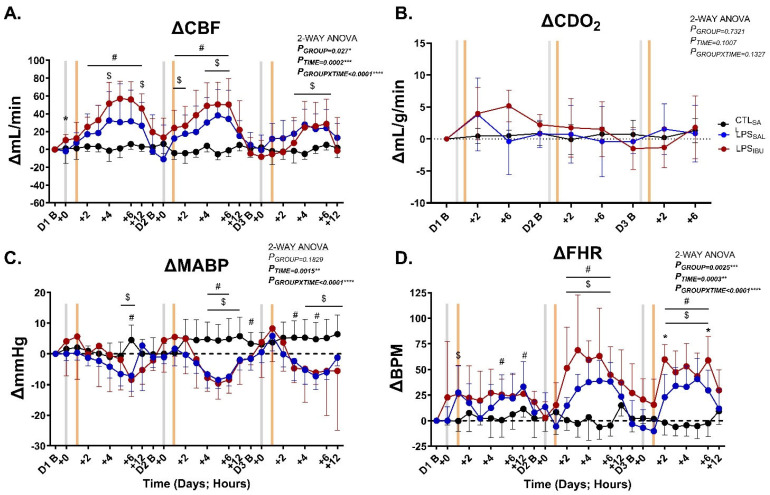
Hemodynamic responses: (**A**) delta change from day 1 baseline (Δ) for right carotid artery blood flow (CBF), (**B**) Δ change of cerebral oxygen delivery (CDO_2_), (**C**) Δ change of mean arterial blood pressure (MABP) and (**D**) Δ change of fetal heart rate (FHR). Hourly epochs were analysed throughout experimental days (**D**) 1–3 at baseline (before LPS/saline infusions [grey bar]) and throughout the first 12 h after LPS/saline infusions. The orange bar denotes the timing of saline or ibuprofen treatment, which was 1 h after the LPS/saline infusion. Values are mean ± SD. CTL_SAL_: *n* = 9; LPS_SAL_: *n* = 8; LPS_IBU_: *n* = 8. Significant group differences were assessed by Tukey’s multiple comparisons test, and are indicated as follows: CTL_SAL_ vs. LPS_SAL_: ^$^ *p* < 0.05; CTL_SAL_ vs. LPS_IBU_: *^#^ p* < 0.05; LPS_SAL_ vs. LPS_IBU_: * p < 0.05.

CBF increased in LPS_SAL_ and LPS_IBU_ fetuses, compared to CTL_SAL_ fetuses, after LPS infusions (Figure 2A), though CBF increased consistently in LPS_IBU_ fetuses between 2 and 12 h after the first LPS dose and at 1 to 6 h following the second dose. There were no differences in CBF between the LPS_IBU_ and LPS_SAL_ groups throughout the experimental period.

CDO_2_ was unchanged following LPS exposure and ibuprofen administration and was not different between groups (Figure 2B).

After the first LPS infusion, MABP was reduced at 5–6 h in LPS_SAL_ and at 6 h in LPS_IBU_ fetuses compared to CTL_SAL_ (Figure 2C), and FHR was significantly higher at 1, 5 and 12 h in LPS_IBU_ fetuses compared to CTL_SAL_ (Figure 2D). After the second and third LPS infusions, the reduction in MABP and rise in FHR was more prominent, occurring between 2 and 6 h after each LPS infusion in both LPS-exposed groups. There was a greater rise in FHR in LPS_IBU_ fetuses compared to LPS_SAL_ fetuses at 2 and 6 h after the third LPS infusion (*p* = 0.009 and 0.043, respectively).

### 2.4. Fetal Blood Gases and Metabolites

**Figure 3:**
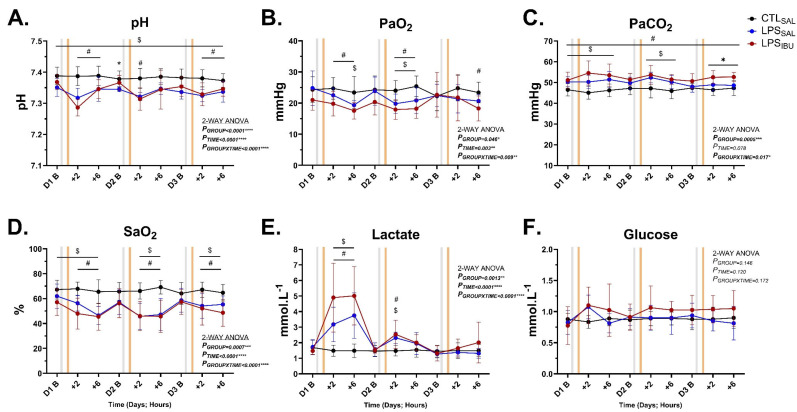
Blood gas measurements of (**A**) pH, (**B**) arterial partial pressure of oxygen (PaO_2_), (**C**) arterial partial pressure of carbon dioxide (PaCO_2_), (**D**) arterial oxygen saturation (SaO_2_), arterial (**E**) lactate and (**F**) glucose. Arterial blood samples were collected on experimental days (**D**) 1–3 at baseline (before LPS/saline infusions [grey bar]) and at 2 and 6 h after LPS/saline infusions. The orange bar denotes the timing of saline or ibuprofen treatment, which was at 1 h after the LPS/saline infusion. Values are mean ± SD. CTL_SAL_: *n* = 9; LPS_SAL_: *n* = 8; LPS_IBU_: *n* = 8. Significant group differences assessed by Fisher’s LSD test for multiple comparisons and are indicated as follows: CTL_SAL_ vs. LPS_SAL_: ^$^ *p* < 0.05; CTL_SAL_ vs. LPS_IBU_: *^#^ p* < 0.05; LPS_SAL_ vs. LPS_IBU_: * *p* < 0.05.

Escalating doses of LPS decreased arterial pH, PaO_2_ and SaO_2_ and increased arterial PaCO_2_ and lactate concentrations (Figure 3A–E). The pH was lower in LPS_SAL_ fetuses compared to CTL_SAL_ fetuses throughout the experimental timeline, while pH in LPS_IBU_ fetuses was lower than CTL_SAL_ from 2 and 6 h after each the first and 3rd LPS infusion (*p* < 0.05). At day 2 baseline, the pH of LPS_IBU_ fetuses was higher than LPS_SAL_ fetuses.

PaO_2_ in LPS_SAL_ fetuses was lower compared to CTL_SAL_ 6 h after the first LPS infusion and at 2 and 6 h after the second LPS infusion, while PaO_2_ in LPS_IBU_ fetuses was significantly lower than CTL_SAL_ at 2 and 6 h after the first and second LPS infusions and 6 h after the third LPS infusion (all *p* < 0.05; Figure 3B). PaCO_2_ levels in LPS_IBU_ fetuses were significantly higher than in the CTL_SAL_ fetuses throughout the experiment. PaCO_2_ levels in LPS_SAL_ fetuses were higher than CTL_SAL_ fetuses at 2 and 6 h after the first and second LPS infusions, as well as at day 1 baseline (Figure 3C). At 2 and 6 h after the third LPS infusion, PaCO_2_ level in LPS_IBU_ fetuses was significantly higher than in LPS_SAL_ fetuses (Figure 2C). SaO_2_ was lower in both LPS_SAL_ and LPS_IBU_ fetuses 2 and 6 h after each of the daily LPS infusions (all *p* < 0.05 compared to CTL_SAL_; Figure 3D). Lactate was higher at 2 and 6 h after the first LPS infusion and at 2 h after the second LPS infusion in both the LPS_SAL_ and the LPS_IBU_ groups (Figure 3E). No differences in arterial glucose levels were found between groups (Figure 3F).

At the end of experiment (48 h after last saline/IBU dose), PaCO_2_ was significantly higher in LPS_IBU_ fetuses, compared to CTL_SAL_ (*p* = 0.038) (Table 1), but arterial pH, PaO_2_, SaO_2_, lactate and glucose levels were not different between groups.

### 2.5. PGE_2_ Levels

**Figure 4:**
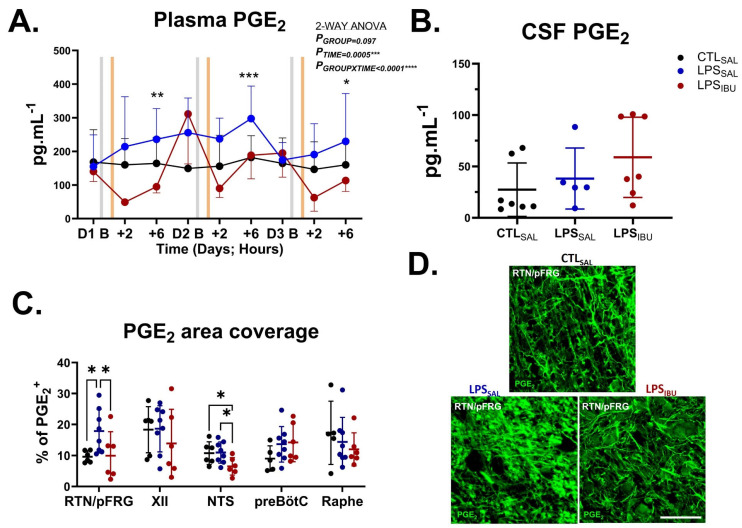
Plasma, CSF and brainstem PGE_2_. (**A**) Concentration of PGE_2_ (pg.mL^−1^) in arterial plasma samples collected periodically throughout the experiment. Arterial plasma samples were collected on experimental days (**D**) 1–3 at baseline (before LPS/saline infusions [grey bar]) and at 2 and 6 h after LPS/saline infusions. The orange bar denotes the timing of saline or ibuprofen treatment, which was at 1 h after the LPS/saline infusion. Significant group differences were assessed by Fisher’s LSD test for multiple comparisons and are indicated as follows: * *p* < 0.05, ** *p* < 0.01, *** *p* < 0.005; LPS_SAL_ vs. LPS_IBU_. CTL_SAL_: *n* = 6; LPS_SAL_: *n* = 8; LPS_IBU_: *n* = 6. (**B**) Concentration of PGE_2_ (pg.mL^−1^) in cerebrospinal fluid (CSF) collected at post-mortem. (**C**) % area coverage of PGE_2_^+^ immunostaining in brainstem respiratory centres. Significant differences are indicated as * *p* < 0.05. Values are mean ± SD. (**D**) Representative PGE_2_ immunofluorescent staining within the RTN/pFRG of the brainstem. Scale bar is 20 µm.

**Figure 5:**
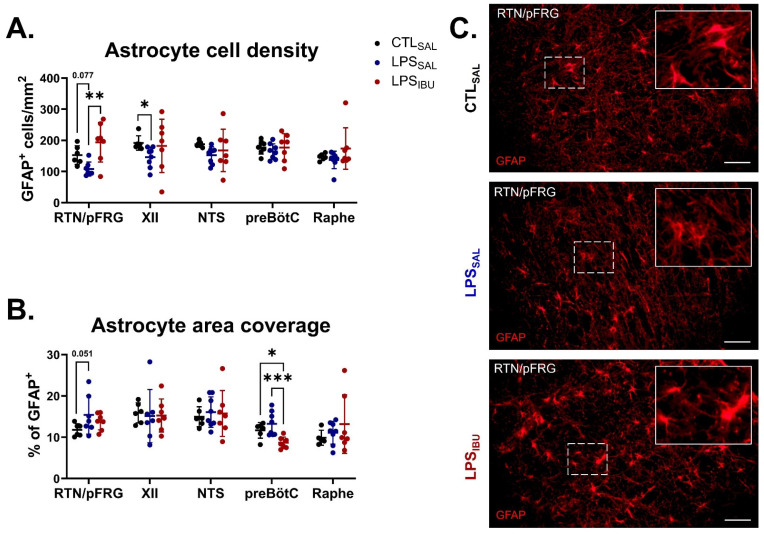
GFAP immunostaining for astrocytes in brainstem respiratory centres. (**A**) Cell density and (**B**) area coverage analysis of GFAP immuno-positive staining in brainstem regions. Values are mean ± SD. Significant differences are indicated as * *p* < 0.05, ** *p* < 0.01 and *** *p* < 0.001. (**C**) Representative GFAP immunofluorescent staining within the RTN/pFRG of the brainstem at 40× magnification. Scale bars are 50 µm.

PGE_2_ levels were assessed within the systemic circulation, the CSF, and key respiratory centres of the brainstem (Figure 4). Circulating PGE_2_ levels at baseline were not different between groups. LPS exposure increased plasma PGE_2_ levels, which peaked at 6 h after the second LPS infusion, though this did not reach statistical significance (*p* = 0.062 LPS_SAL_ vs. CTL_SAL_; Figure 4A). Ibuprofen treatment decreased circulating PGE_2_ levels to levels below CTL_SAL_ and was significantly lower than in LPS_SAL_ fetuses at 6 h after the first LPS infusion and 2 h after the second and third LPS infusions (*p* < 0.05; Figure 5A). CSF PGE_2_ levels were not different between groups (Figure 4B).

PGE_2_ immunostaining was increased in the retrotrapezoid/parafacial respiratory group (RTN/pFRG) in LPS_SAL_ fetuses, compared to both CTL_SAL_ (*p* = 0.026) and LPS_IBU_ fetuses (*p* = 0.031), but did not differ between CTL_SAL_-treated and LPS_IBU_-treated fetuses (Figure 4C,D). In the nucleus tractus solitarius (NTS), LPS_IBU_ fetuses had decreased PGE_2_ immunostaining compared to CTL_SAL_ (*p* = 0.041) and LPS_SAL_ fetuses (*p* = 0.024). No differences in PGE_2_ immunostaining were found between groups in the hypoglossal nucleus (XII), preBötzinger complex (preBötC), or raphe nucleus.

### 2.6. Microglia and Astrocyte Assessments

In LPS_SAL_ fetuses, glial fibrillary acidic protein (GFAP)^+^ cell density in the XII nucleus was reduced, compared to CTL_SAL_ fetuses (*p* = 0.026). Moreover, a trend towards reduced GFAP^+^ cell density also occurred in the RTN/pFRG (*p* = 0.077; Figure 5A). GFAP^+^ area coverage tended to be higher in the RTN/pFRG in LPS_SAL_ fetuses, compared to CTL_SAL_ fetuses (*p* = 0.051; Figure 5B). In LPS_IBU_ fetuses, GFAP^+^ cell density in the RTN/pFRG was significantly higher than in LPS_SAL_ fetuses (*p* = 0.002; Figure 5A), but was not different from that in CTL_SAL_ fetuses. Similarly, the GFAP^+^ cell density of LPS_IBU_ fetuses in the XII nucleus was also not different from that in CTL_SAL_ fetuses. Ibuprofen reduced the GFAP^+^ area coverage in the preBötC of LPS_IBU_ fetuses, compared to both CTL_SAL_ and LPS_SAL_ fetuses (*p* = 0.026 and 0.0009, respectively; Figure 5B). No differences in GFAP^+^ cell density or area coverage between groups were found in the NTS or raphe nucleus.

**Figure 6:**
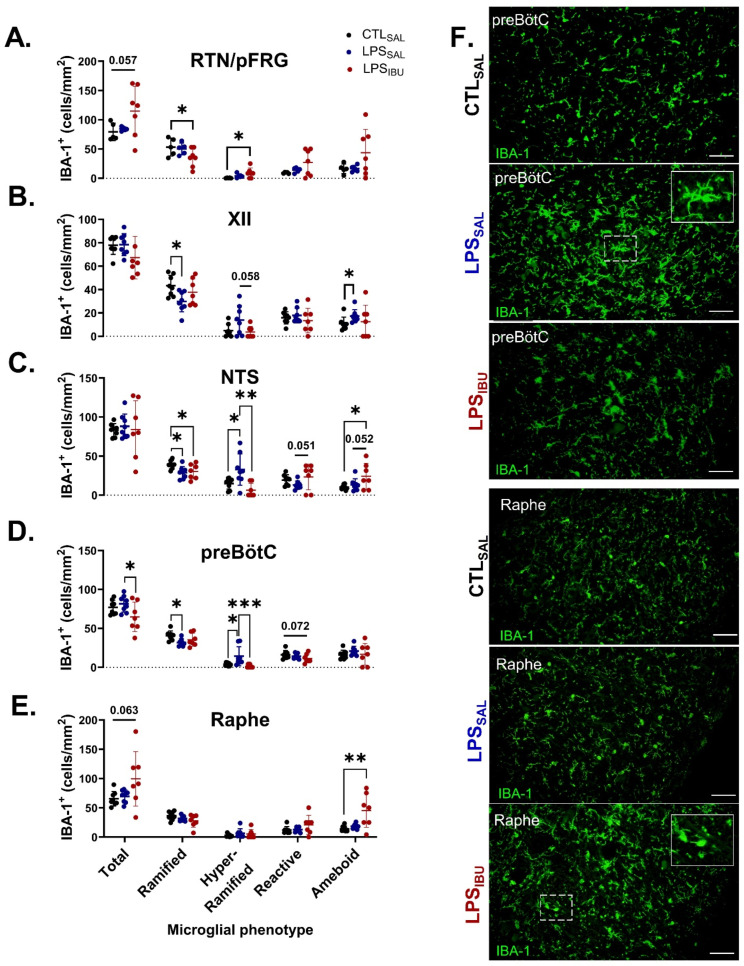
IBA-1 immunostaining for microglia in brainstem respiratory centres. (**A**–**E**) Cell density analysis of IBA-1 immuno-positive staining in brainstem regions. Values are mean ± SD. Significant differences are indicated as * *p* < 0.05, ** *p* < 0.01, *** *p* < 0.005. (**F**) Representative IBA-1 immunofluorescent staining within the preBötC and raphe of the brainstem at 40× magnification. Scale bars are 50 µm.

In the RTN/pFRG nucleus, LPS exposure did not alter microglial density. However, LPS_IBU_ fetuses tended to increase total microglia (*p* = 0.057). Moreover, the density of hyper-ramified microglia was significantly higher compared to CTL_SAL_ (*p* = 0.027), concordant with a decrease in the density of ramified microglia (*p* = 0.045; Figure 6A). LPS exposure decreased the density of ramified microglia in the XII, NTS and preBötC (Figure 6B–D). In the XII nucleus, specifically in LPS_SAL_ fetuses, there was a higher density of ameboid microglia compared to that in CTL_SAL_ (*p* = 0.039), and a trend towards a higher density of hyper-ramified microglia, compared to LPS_IBU_ fetuses (*p* = 0.058; Figure 6B). In the NTS, LPS_SAL_ fetuses had an increased density of hyper-ramified microglia, compared to CTL_SAL_ fetuses (*p* = 0.015 Figure 6C). Ibuprofen reduced the hyper-ramified microglia to control levels; however, LPS_IBU_ fetuses had increased densities of reactive (compared to LPS_SAL_) and ameboid (compared to CTL_SAL_ and LPS_SAL_) microglia (Figure 6C). Similarly, in the preBötC, LPS_SAL_ fetuses had increased density of hyper-ramified microglia, compared to CTL_SAL_ fetuses (*p* = 0.029), with ibuprofen restoring the density of hyper-ramified microglia in the preBötC (*p* = 0.0002); this resulted in a reduction in total microglia density and reactive microglia in LPS_IBU_ fetuses (Figure 6D). In the raphe nucleus, there was an increase in the number of ameboid microglia in LPS_IBU_ fetuses and a trend towards increased total microglial density (*p* = 0.003 and 0.063, respectively; Figure 6E).

## 3. Discussion

Antenatal exposure to infection/inflammation is associated with fetal [14,15] and newborn [12,16,17] respiratory depression. We previously showed that a course of escalating doses of intravenous LPS inhibits FBM incidence, concurrent with upregulation of PGE_2_ within brainstem respiratory centres [13]. In this study, we aimed to determine whether ibuprofen, a non-selective COX inhibitor, could restore FBMs though the reduction of systemic and brainstem respiratory centre PGE_2_ levels. In contrast to our hypothesis, and despite ibuprofen reducing the LPS-induced increase in circulating and brainstem PGE_2_, ibuprofen did not restore FBMs in LPS-exposed fetuses compared to controls. Moreover, while ibuprofen reduced markers of astrocytosis and microgliosis in the preBötC, there were increased numbers of morphologically activated microglia and total astrocytes in the raphe nucleus, NTS and RTN/pFRG of the brainstem. Overall, this suggests that ibuprofen, at the dosage used, had limited effects on histological markers of brainstem gliosis. Collectively, these data indicate that ibuprofen does not restore inflammation-mediated inhibition of FBMs, and that LPS-induced inhibition of FBMs is not exclusively driven by high PGE_2_ levels in the circulation and brainstem respiratory centres.

Given that the upregulation of PGE_2_ has been associated with the suppression of breathing in infants exposed to inflammation/infection [3,12], we utilised ibuprofen to determine whether inhibition of PGE_2_ would restore FBM. Ibuprofen is a commonly used drug in neonatal medicine, predominately utilised for the treatment of hemodynamically significant patent ductus arteriosus. Ibuprofen is a non-selective COX inhibitor that inhibits the conversion of arachidonic acid to prostaglandin H_2_, thus preventing downstream conversion to several prostaglandins, including PGE_2_. Ibuprofen works promptly when given intravenously, but is metabolised and eliminated rapidly, within a 24 h period [18]. Ibuprofen has been shown to cross the blood–brain barrier [19] and to reduce neuroinflammation in neonatal [20,21,22] and adult [23] animal studies investigating neuroinflammatory conditions. In our study, we show that despite reducing PGE_2_ expression systemically and within key respiratory centres (NTS and RTN/pFRG), ibuprofen did not restore FBMs, suggesting it is not the dominant promoter of FBM inhibition following intrauterine inflammation.

The likely reason why ibuprofen did not restore FBMs is the potential involvement of other pro-inflammatory mediators induced in parallel to PGE_2_, and having a stronger influence on inflammation-induced inhibition of breathing. In this experimental model we showed that progressive LPS-induced inflammation causes an increase in systemic and central pro-inflammatory cytokines, including IL-1β, TNF, IL-8 and IL-6 [24,25]. Pro-inflammatory mediators, particularly IL-1β, can affect neuronal signalling within the brainstem respiratory centres to depress respiratory behaviour [26,27]. Indeed, IL-1β induces mPGES-1 (inducible microsomal Prostaglandin-1) and subsequent PGE_2_ release within brainstem respiratory centres and is associated with respiratory inhibition in mice and humans [12,28]. However, IL-1β can directly increase excitatory signalling in the brainstem and depress inhibitory synaptic transmission [29], and alter chemosensory responses to hypercapnia and hypoxia [30,31]. TNF and IL-6 can also modulate neuronal function within the central nervous system by potentiating excitatory signalling and depressing inhibitory transmission, particularly within the preBötC [32]. It is possible that these proinflammatory cytokines, independent of PGE_2_, are exerting a stronger influence on inflammation-induced respiratory inhibition. Indeed, despite the reduction in PGE_2_ within the RTN/pFRG and NTS with ibuprofen in LPS-exposed fetuses compared to controls, we observed increased numbers of astrocytes (RTN/pFRG) and a shift in microglial morphology from a more quiescent state towards an activated state within the RTN/pFRG and NTS. Overall, these data suggest there is limited relationship between ibuprofen-induced PGE_2_ inhibition and histological markers of brainstem gliosis.

Ibuprofen administration resulted in higher carotid blood flow and heart rate in LPS-exposed fetuses. These effects are likely consequences of the haemodynamic effects of ibuprofen on the fetal circulation. PGE_2_ is a major regulator of ductus arteriosus patency [5]. We were unable to confirm whether ibuprofen affected patency of the ductus arteriosus, but we did observe greater heart weights in LPS_IBU_ fetuses. It is possible that ibuprofen-induced narrowing of the ductus arteriosus promoted cardiac hypertrophy by means of increasing the pulmonary perfusion, which, in turn, increased ventricular pressure. Another interesting finding was that LPS_IBU_ had higher PaCO_2_ compared to LPS_SAL_ fetuses. The increased CBF observed in ibuprofen-treated fetuses could also be explained by an increased arterial CO_2_ concentration in the LPS_IBU_ group, which may have promoted greater vasodilation of cerebral arteries and arterioles.

Hypercapnia, in itself, stimulates FBMs [33]. Despite the higher PCO_2_ after ibuprofen, the FBM incidence did not change, suggesting that under these conditions, a moderate increase in arterial CO_2_ concentration (~5 mmHg above baseline) has limited effects on FBMs. Interestingly, we did observe an increase in the proportion of accentuated or “deep” breathing in ibuprofen-treated fetuses. The brainstem contains central chemoreceptors that drive respiration in a CO_2_-dependant manner to increase inspiratory depth [34,35]. This may explain the increase in FBM amplitude in the LPS_IBU_ group.

Future directions for study in this area include investigating different doses of ibuprofen or other, more selective, PGE_2_ inhibitors, such as indomethacin, and performing a more specific interrogation of the biochemical pathways of fetal breathing inhibition. For example, we were not able to measure the different subtypes of PGE_2_ metabolites or receptors within the brainstem respiratory centres due to species-related limitations with respect to antibodies. PGE_2_ is implicated in a number of neuropathological conditions due to its capacity to bind to several G-protein-coupled receptors [36,37]. These receptors are differentially expressed spatially across brainstem regions, and binding to them results in distinct signalling pathways, all of which can induce damage or dysfunction, and ameliorate or potentiate inflammation and alter breathing. Investigating further the inflammatory pathways that dictate respiratory depression, particularly those involved in driving the production of the key inflammatory proteins (e.g., IL-1 and IL-6) associated with exposure to perinatal infection/inflammation and respiratory depression, may reveal future therapeutic targets. Further, the optimal dose of ibuprofen is not known. We used the lowest effective dose which has demonstrated clinical efficacy and safety in humans. Higher doses may be more effective, but whether these have physiological effects, particularly on the patent ductus arteriosus, is not well understood, and warrants further investigation. Indeed, there are limited data on ibuprofen pharmacokinetics and pharmacodynamics during fetal development. Importantly, we were able to demonstrate a reduction in systemic PGE2 at the dose used. Fetal sheep, and not neonatal lambs, were used for this study to eliminate the confounding effects of prostaglandins on the transition at birth and the respiratory-depressive effects of anaesthesia. Moreover, reduced FBM are associated with later diagnosis of antenatal exposure to infection/inflammation [14,15] and respiratory depression in newborns [12,16,17]. Utilising a neonatal model will determine whether systemic inflammation and the subsequent depression of FBMs translate into impaired respiratory function at birth, and whether therapies targeting restoration of fetal breathing movements translate into the newborn paradigm.

## 4. Materials and Methods

### 4.1. Ethics Statement

Experimental procedures were approved by the Monash Medical Centre Animal Ethics Committee, Monash University, Australia (MMCA/2017/13 and MMCA/2018/17) and conducted in accordance with the *Australian code for the care and use of animals for scientific purposes* established by the National Health and Medical Research Council of Australia.

### 4.2. Animal Experimental Procedures

Twenty-five pregnant Border Leicester ewes carrying singletons or twins at a gestational age of 125 ± 1 days (d; term is ∼148 d) were utilised for this study. All methodology pertaining to preoperative procedures, sterile surgery, anaesthesia and analgesia has been described previously [13,24,25]. The fetus was instrumentally monitored with

1. Tracheal catheter to measure fetal breathing movements (FBM);
2. Right brachial arterial catheter to measure mean arterial blood pressure (MABP) and fetal heart rate (FHR), and for sampling arterial blood gases;
3. Right axillary vein catheter to administer postoperative antibiotics, LPS/saline, and ibuprofen;
4. Amniotic catheter for fetal MABP correction from maternal movement;
5. A right carotid artery ultrasonic flow probe (3 mm) to measure carotid arterial blood flow (CBF; Transonic Systems, Ithaca, NY, USA) and fetal heart rate, which was derived from the carotid arterial beat-to-beat interval.

**Figure 7:**
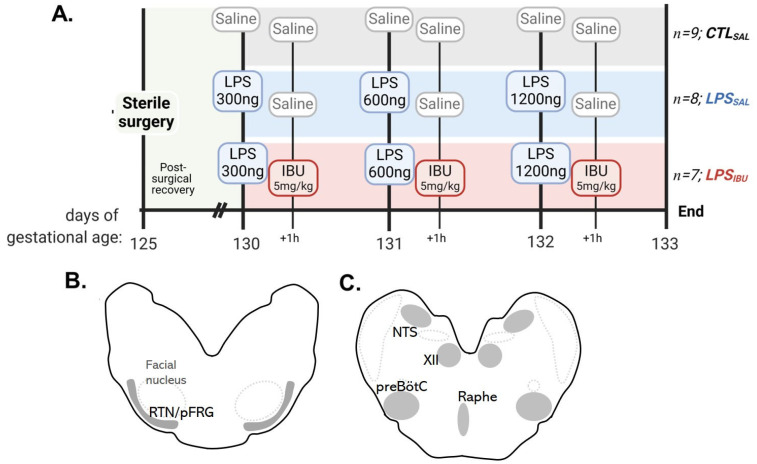
Experimental timeline and brainstem assessment. (**A**) Instrumentally monitored fetuses randomly allocated to groups of CTL_SAL_ fetuses only administered saline; LPS_SAL_ fetuses administered daily-escalated doses of lipopolysaccharide (LPS), and saline 1 h after each LPS dose; and LPS_IBU_ fetuses administered daily-escalated doses of lipopolysaccharide (LPS), with each LPS dose followed by ibuprofen (IBU) 1 h after. Respiratory centres assessed within the brainstem medulla in grey: (**B**) retrotrapezoid/parafacial respiratory group (RTN/pFRG); (**C**) nucleus tractus solitarius (NTS), preBötzinger complex (preBötC), raphe nucleus and the hypoglossal nucleus (XII).

At ~130 ± 1 d fetal sheep were randomly assigned, using an online number generator, to either control and saline (CTL_SAL_, *n* = 9); LPS and saline (LPS_SAL_, *n* = 8); or LPS and ibuprofen (IBU) (LPS_IBU_, *n* = 8). Fetuses assigned to LPS groups received daily intravenous (i.v.) escalating doses of infusions of lipopolysaccharide (LPS; E. Coli O55:B5; Sigma-Aldrich, Australia) at doses of 300 ng on experimental day 1, 600 ng on experimental day 2 and 1200 ng on experimental day 3; these were diluted in 2 mL saline to induce a progressive systemic inflammatory response, as described previously [24]. Ibuprofen-treated fetuses (LPS_IBU_) received an i.v. bolus of ibuprofen (5 mg.kg^−1^.d^−1^; diluted in sterile water; 2 mL) 1 h after each LPS infusion. This dose of ibuprofen was chosen since it is the lowest dose given to human preterm infants for treatment of a patent ductus arteriosus. CTL_SAL_ fetuses received isovolumetric saline (2 mL) intravenously at the time of LPS/IBU interventions. Ewes and fetuses were euthanised at 135 d (4 days after the start of infusions) with an intravenous overdose of pentobarbital sodium to the ewe (100 mg.kg^−1^; Lethabarb; Virbac, Milperra, NSW, Australia) (Figure 7A).

### 4.3. Physiology Measurements and Sample Collection

Fetal catheters were connected to pressure transducers (DTX Plus, BD Medical Systems, North Ryde, NSW, Australia) and the carotid arterial flow probe connected to a flow-meter, and all physiological data were continuously digitally recorded using a PowerLab A-D converter and stored using LabChart pro software (version 8, ADInstruments, Bella Vista, NSW, Australia). All physiological analyses were performed offline by an investigator who was blinded to the treatment group. One-hour epochs of physiological recordings were sampled on days 1, 2 and 3 at baseline (defined as 1 h prior to LPS dose); +0 (at LPS dose); +1; +2; +3; +4; +5; +6; and +12 h (relative to LPS dose). Within each one-hour epoch, all periods of breathing and apnoea were individually selected and recorded. FBM were defined as consistent breathing movements with tracheal pressures less than −1.5 mmHg. Multiple FBM characteristics were analysed, including (i) incidence, the total time spent breathing expressed as the % of time FBMs occurred per hour; (ii) duration, the average duration of FBMs (s) per hour; (iii) frequency, the average number of breaths/second during an episode of FBMs per hour; (iv) amplitude, average depth of individual breaths (mmHg) per hour; and (v) accentuated breathing, the total time spent breathing with an average amplitude > 5 mmHg, expressed as the % of time FBMs occurred per hour [38]. Average CBF, MABP and FHR during apnoeic periods were calculated at the same timepoints. Cerebral oxygen delivery (CDO_2_) was calculated using CBF and blood gas measurements [39]:

(1) $${CDO}_{2}=\frac{{CBF}_{average}\times \left(arterial\;oxygen\;content\right)}{brain\;weight\;(kg)}$$

where arterial oxygen content is calculated as described in the following, in which Hb is the hemoglobin concentration (g/dL):

(2) $$Arterial\;oxygen\;content=(\left[1.3\times Hb\times \frac{SaO_{2}}{100}\right]+\left[0.03\times {PaO}_{2}\right])$$

Daily arterial blood samples were collected to monitor fetal blood biochemistry and for plasma collection at baseline, +2 and +6 h after each saline or LPS infusion. Blood gas values for pH, partial pressure of carbon dioxide (PaCO_2_), partial pressure of oxygen (PaO_2_), oxygen saturation (SaO_2_), and lactate were measured using an ABL90 Flex Pus analyser (Radiometer, Brønshøj, Denmark). Blood plasma samples were obtained by centrifuging the blood sample at 3000× *g* for 10 min; the plasma collected was then stored at −80 °C.

At post-mortem, cerebrospinal fluid (CSF) was collected from a cisternae magna puncture and centrifuged, and then the supernatant was collected and immediately frozen in liquid nitrogen. All organs were weighed. The whole brainstem was collected and immersion-fixed in 10% neutral buffered formalin for 6 days at 4 °C prior to paraffin processing and embedding. Serial brainstem sections were cut at an 8 µm thickness. Brainstem respiratory centres were identified using the Michigan State sheep brain atlas [40] and a rat brain atlas [41]. The putative retrotrapezoid/parafacial respiratory group (RTN/pFRG) area was identified ventral to the facial nucleus in sections of the medulla oblongata and inferior cerebellar peduncle. In the same section, the raphe nucleus (pallidus) was identified as medial to the RTN/pFRG. The nucleus tractus solitarius (NTS) and the putative preBötzinger complex (preBötC) were identified at a level caudal to the cerebellar peduncle; all were above the central canal. Anatomical landmarks for identifying the preBötC included the NTS and hypoglossal nucleus (XII) (Figure 7B,C).

### 4.4. Immunohistochemistry

Brainstem sections were immunolabelled for PGE_2_, ionized calcium binding adaptor molecule-1 (IBA-1; microglia) and glial fibrillary acidic protein (GFAP; astrocytes), as previously reported [13]. Briefly, sections were dewaxed and subjected to antigen retrieval with Proteinase K (Sigma-Aldrich, Bayswater, Australia) for PGE_2_ immunolabelling, and heat-mediated citrate buffer for IBA-1 and GFAP immunolabelling. Sections were blocked and then incubated with the primary antibodies anti-rabbit PGE_2_ (1:200; Abcam, Melbourne, Australia), anti-mouse GFAP (1:500; Abcam, Australia), and anti-rabbit IBA-1 (1:500; WAKO Chemicals, Japan) overnight, at 4 °C. Sections were washed, then incubated with a respective secondary antibody: anti-rabbit Alexa Fluor 488 for PGE_2_ (1:150) and IBA-1 (1:200), and anti-mouse Alexa Fluor 594 for GFAP (1:200); all were sourced from Jackson Immuno Research, USA. Sections were also incubated with the nuclear stain HOESCHT (1:1000 dilution in 1x PBS; Invitrogen, Waltham, MA, USA) for 5 min, then washed for 5 min in PBS. Sections were cover-slipped using DAKO anti-fade fluorescent mounting medium (Agilent Technologies, Mulgrave, Australia). In the PGE_2_ immunostaining, four samples from the CTL_SAL_ and two samples from the IBU_LPS_ group could not be assessed

### 4.5. Fluorescent Imaging and Quantitative Analysis

All fluorescent images were obtained at 40× magnification using an Olympus BX50 microscope and Cell Sense imaging software (Version 2.3, Olympus, Notting Hill, Australia). A total of four non-overlapping fields of view (FOV; 200 µm × 200 µm) were taken per respiratory centre per subject. No staining was observed in negative controls where the primary antibody was omitted. Slides and images were coded, and the assessor was blinded to the group allocation. The % area coverage of PGE_2_+ and GFAP+ labelling was measured using a set intensity threshold (FIJI; ImageJ, version 2.00, NIH Image, Bethesda, MD, USA). The total number of GFAP+ astrocytes and IBA-1+ microglia in each FOV from each respiratory centre was counted and expressed as mean total number of cells/mm^2^. Microglia (IBA-1 + cells) showing ramified (small cell body with >1 branching process) or amoeboid morphology (large cell bodies, with ≤1 branching process) were included in our assessment [23].

### 4.6. Plasma and CSF PGE_2_

To determine PGE_2_ levels within CSF and blood plasma, a monoclonal PGE_2_ enzyme-linked immunosorbent assay (ELISA) kit (Cayman Chemicals, Ann Arbor, MI, USA) was utilised according to the manufacturer’s instructions and the results were expressed as % binding activity. Three replicates were used per sample at each time point. The intra-assay and inter-assay coefficients of variability (%CV) were set to <20%. Due to technical difficulties relating to sample collection, two CSF and three plasma samples from CTL_SAL_, three CSF samples from LPS_SAL_, and one CSF and two plasma samples from LPS_IBU_ were not included in the analyses of PGE_2_ levels.

### 4.7. Statistical Analysis

Average physiological measurements (FBM characteristics, CBF, CDO_2_, MABP and FHR) at baseline (i.e., 1 h prior to first LPS infusion) were compared between groups using a one-way ANOVA (for parametric data) or the Kruskal–Wallis test (for non-parametric data). Normality was assessed using the Shapiro–Wilk test. Due to small but non-significant differences in the baseline FBM characteristics (except for FBM incidence), CBF, CDO_2_, MABP and FHR data are expressed as delta change from day one baseline. Physiological data were analysed using a mixed-effects two-way ANOVA with repeated measures. The independent variables assessed were group (*P*_GROUP_) and time of measurement (*P*_TIME_). Where there was a significant interaction, a post hoc analysis with Fisher’s least significant difference (LSD) test was used to determine differences between groups at specific time points. Animal characteristics, immunohistochemical data, and CSF PGE_2_ data were analysed using a one-way ANOVA (for parametric data) or the Kruskal–Wallis test (for non-parametric data); normality was assessed using the Shapiro–Wilk test. Significant effects were the subjects of follow-up to identify differences between groups using Fisher’s LSD test or an uncorrected Dunn’s test, for parametric and non-parametric data, respectively. All analyses were performed using GraphPad Prism v9 (GraphPad Software, La Jolla, CA, USA). Data are presented as mean ± standard deviation (SD). A *p* < 0.05 determined statistical significance.

## 5. Conclusions

We found that reducing systemic and localised PGE_2_ within key brainstem respiratory centres using intravenous ibuprofen did not mitigate the inflammation-induced depression of FBMs in late-gestation fetal sheep. The mediators of breathing inhibition associated with intrauterine inflammation are likely to involve a complex interplay of pro-inflammatory mediators beyond PGE_2_. Understanding their effects on dynamic network connectivity in brainstem respiratory centres will be key to elucidating why respiratory failure occurs in preterm infants exposed to intrauterine inflammation.

## Figures and Tables

**Table 1:** Fetal characteristics at end of experiment and post-mortem.

	CTL_SAL_	LPS_SAL_	LPS_IBU_	
Number (n)	9	8	8	
Male/Female	7:2	7:1	5:3	
Single/Twin	6:3	5:3	8:0	***p-Value***
Body weight (kg)	4.55 ± 0.60	4.56 ± 0.49	4.84 ± 0.38	0.196
Brain weight (g)	51.47 ± 3.70	48.77 ± 3.83	47.73 ± 3.39	0.131
Brain/Body ratio	11.40 ± 1.77	10.75 ± 0.77	9.88 ± 0.44	0.088
Heart weight (g)	31.98 ± 5.75	32.94 ± 4.97	40.20 ± 3.07 **^##^	0.004
Heart/Body ratio	7.06 ± 1.07	7.26 ± 1.03	8.37 ± 1.02 ^#^	0.046
Lungs (g)	180.00 ± 27.17	156.00 ± 17.00	178.83 ± 41.85	0.219
Lungs/Body Ratio	39.80 ± 5.63	34.34 ± 3.05	37.02 ± 8.68	0.104
Liver (g)	131.10 ± 30.06	134.40 ± 21.41	142.38 ± 23.48	0.433
Right Kidney (g)	12.39 ± 3.03	12.66 ± 1.50	13.60 ± 1.94	0.537
Blood gases at end of experiment				
pH	7.4 ± 0.0	7.3 ± 0.0	7.3 ± 0.0	0.136
PaO_2_ (mmHg)	24.1 ± 2.6	23.4 ± 1.7	21.3 ± 4.3	0.192
PaCO_2_ (mmHg)	47.2 ± 3.5	48.2 ± 1.9	51.2 ± 3.3 ^#^	0.039
SaO_2_ (%)	64.6 ± 7.4	61.7 ± 5.7	60.6 ± 14.8	0.716
Lactate (mmol.L^−1^)	1.5 ± 0.4	1.0 ± 0.2	1.5 ± 0.8	0.161
Glucose (mmol.L^−1^)	0.9 ± 0.1	0.9 ± 0.2	1.2 ± 0.3	0.056

Data expressed as mean ± SD and analysed by one-way ANOVA for parametric data or the Kruskal–Wallis test for non-parametric data; post hoc tests followed up with Tukey’s multiple comparisons test. Significant group differences are in bold and indicated as follows: CTL_SAL_ vs. LPS_IBU_: *^#^ p* < 0.05, *^##^ p* < 0.01, LPS_SAL_ vs. LPS_IBU_: ** *p* < 0.01.

**Table 2:** Baseline fetal breathing, cardiovascular, and cerebral physiology data: Average baseline data, obtained one hour prior to the first LPS/saline infusion, for fetal breathing movement characterisation, carotid blood flow (CBF), cerebral oxygen delivery (CDO_2_), mean arterial pressure (MABP) and fetal heart rate (FHR) parameters. Statistical analysis using one-way ANOVA for parametric data or the Kruskal–Wallis test for non-parametric data, demonstrating no baseline differences between groups. Data are mean ± SD.

	Group			
	CTL_SAL_	LPS_SAL_	LPS_IBU_	*p*-Value
Breathing incidence (%)	44.0 ± 16.2	38.6 ± 26.4	28.9 ± 19.9	0.346
Breathing duration (s)	75.5 ± 70.2	192.8 ± 171.7	145.6 ± 85.0	0.152
Breathing frequency (breaths/s)	2.5 ± 0.2	4.0 ± 3.4	2.7 ± 1.2	0.967
Breathing amplitude (mmHg)	6.6 ± 2.9	5.8 ± 2.8	4.2 ± 1.5	0.230
Accentuated breathing (% of time spent breathing)	7.5 ± 4.2	34.4 ± 30.6	10.3 ± 10.0	0.233
CBF (mL/min)	72.3 ± 9.8	90.7 ± 26.2	64.0 ± 16.1	0.085
CDO_2_ (mL/g/min)	11.6 ± 1.9	16.5 ± 5.6	12.4 ± 2.5	0.099
MABP (mmHg)	39.6 ± 7.0	44.2 ± 2.9	43.5 ± 6.3	0.230
FHR (bpm)	155.0 ± 14.4	146.2 ± 31.5	158.1 ± 12.6	0.624

## Data Availability

Data is provided within the manuscript. The datasets used during the current study are available from the corresponding author upon reasonable request.

## Abbreviations

The following abbreviations are used in this manuscript:

%	Percent
°C	Degrees Celsius
µm	Micrometre
ANOVA	Analysis of variance
CBF	Carotid blood flow
CDO_2_	Cerebral oxygen delivery
COX	Cyclooxygenase
CSF	Cerebral spinal fluid
CTL	Control
CV	Coefficient of variability
d/D	Days
dGA	Days gestational age
ELISA	Enzyme-linked immunosorbent assay
FBM	Fetal breathing movement
FDA	Federal drug administration
FHR	Fetal heart rate
GFAP	Glial fibrillary acidic protein
g	Grams
h	Hour
IBA	Ionized calcium binding adaptor molecule
IBU	Ibuprofen
IL	Interleukin
i.v.	Intravenous
Kg	Kilogram
LPS	Lipopolysaccharide
MABP	Mean arterial blood pressure
mg	Milligram
Min	Minute
mm	Millimetre
mmHg	Millimetres of mercury
mPGES-1	Microsomal prostaglandin E synthase-1
n	Number of
NTS	Nucleus tractus solitarius
PGE_2_	Prostaglandin E_2_
PaCO_2_	Arterial partial pressure of carbon dioxide
PaO_2_	Arterial partial pressure of oxygen
PBS	Phosphate-buffered saline
PDA	Patent ductus arteriosus
pFRG	Parafacial respiratory group
preBötC	preBötzinger
RTN	Retrotrapezoid
s	Second
SD	Standard deviation
SAL	Saline
SO_2_	Arterial oxygen saturation
TNF	Tumor necrosis factor
XII	Hypoglossal nucleus
